# Characterization of Microencapsulated *Thymus schimperi* Essential Oil Prepared by Spray and Freeze‐Drying Using Gum Arabic as Carrier Material

**DOI:** 10.1002/fsn3.70604

**Published:** 2025-07-13

**Authors:** Yisgedu Asres, Ariaya Hymete, Habtamu Admassu, Amare Ayalew

**Affiliations:** ^1^ Department of Chemical Engineering Addis Ababa Science and Technology University Addis Ababa Ethiopia; ^2^ Department of Food Engineering Debre Birhan University Debre Birhan Ethiopia; ^3^ Department of Pharmaceutical Chemistry and Pharmacognosy, School of Pharmacy Addis Ababa University Addis Ababa Ethiopia; ^4^ Food Process Engineering Program Addis Ababa Science and Technology University Addis Ababa Ethiopia; ^5^ Biotechnology and Bioprocess Center of Excellence Addis Ababa Science and Technology University Addis Ababa Ethiopia; ^6^ Department of Chemistry Debre Birhan University Debre Birhan Ethiopia

**Keywords:** freeze drier, gum Arabic, microencapsulation, morphology, spray drier, *Thymus schimperi*

## Abstract

The process of microencapsulation is used to coat essential oils that are vulnerable to oxidation, dampness, UV light, and high temperatures. It serves to shield the active chemicals from the environment while boosting the core material's controlled release, stability, and solubility. This work used spray and freeze‐drying procedures to microencapsulate essential oil extracted from *Thymus schimperi* ( *T. schimperi* ) leaves using hydro distillation. The objective of this study was to investigate the impact of gum Arabic as a coating agent on the physicochemical properties of *T. schimperi* essential oil microcapsules through spray and freeze‐drying encapsulation procedures. Water content, surface morphology, solubility, bulk density, tapped density, surface oil, flowability, particle mean diameter, and other physical and chemical properties were examined. The physico‐chemical properties of the microencapsulated samples were examined, including their releasing property, functional group identification, thermal property, and storage stability. The features of microcapsules made by spray drying differ greatly from those made by freeze drying. According to the results, spray drying was shown to have a higher encapsulation efficiency (71.05%) than freeze drying (68.89%). The solubility index of spray‐dried microcapsules was higher than that of freeze‐dried microcapsules. When compared to freeze‐dried encapsulated *T. schimperi* EO, the bulk density of spray‐dried encapsulated EO is lower, whereas the tapped density is reversed. Compared to freeze‐dried microcapsules, spray‐dried microcapsules had a more spherical shape.

## Graphical abstract

This study investigated gum Arabic's impact as a coating on *Thymus schimperi* essential oil microcapsules using spray and freeze‐drying. The research examined various physicochemical properties, including water content, density, and solubility. Spray drying yielded higher encapsulation efficiency (71.05%) than freeze drying (68.89%) and better solubility. Spray‐dried microcapsules were also more spherical, demonstrating significant differences in properties compared to freeze‐dried ones.

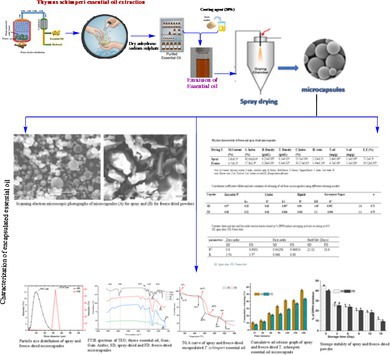

## 1 Introduction

Essential oils are secondary metabolites that are recognized by their unique qualities and naturally occurring scent. Solvent extraction, steam distillation, and cold pressing are common methods used to obtain these oils. They have some of the plant's medicinal qualities as well as the essence of its aroma. The businesses of medicine, pharmaceuticals, cosmetics, and food all employ essential oils (Marques and Borges 2017). They are typically liquids at room temperature and are extremely vulnerable to high temperatures, humidity, and oxygen (Botrel and Oliveira 2014). Numerous essential oils have actions that are bioactive. Because they can disrupt the lipid bilayer of bacteria, which in turn causes the degeneration of the cell membrane and ultimately leads to bacterial death, they have antibacterial activity. Thyme oil is one among the essential oils that have good biological activity due to the presence of a high percentage of thymol, p‐cymene, carvacrol, and γ‐terpinene (Radünz et al. 2020). The primary challenge with essential oils is their susceptibility to volatilization and degradation, which can be caused by high temperature, oxidation, and UV light, leading to a significant loss of their biological activity. Microencapsulation is the most efficient method to protect active components against the surrounding environment (Tomazelli Júnior et al. 2018).

The method of microencapsulation involves enclosing minute particles or droplets of an active substance—such as medications, essential oils, or other bioactive compounds—into a coating or matrix material to create tiny capsules (Burhan et al. 2019). These microcapsules range in size from a few micrometers to many millimeters. In order to maintain the active substance's stability and biological activity, microencapsulation serves to shield it from external elements such as heat, light, oxygen, and moisture (Ogrodowska et al. 2020). Wilkowska et al. (2016) have reported that microencapsulation offers enhanced handling, targeted distribution, and controlled release of the active ingredient. These microcapsules are applied to the food sector as a natural food preservative, flavor enhancer, or functional ingredient. Various encapsulation techniques exist, contingent upon the properties of the core and wall matrix, the intended microcapsule size, and additional variables such as resistance to high temperature. The two encapsulation techniques that are most frequently employed are spray drying and freeze drying (Gharsallaoui and Chambin 2007). The mechanism of freeze‐drying is the sublimation of a frozen substance to dehydrate it. Because substances are not subjected to high temperatures, the biological and nutritional properties of the freeze‐dried items are preserved. Conversely, spray drying is an encapsulation method that involves continuously processing feed in a hot drying solution to transform it from a fluid condition into a powder. The spray drying method works best for products that behave like liquids and maximizes heat transfer. For certain heat‐sensitive industrial items, spray drying is also preferred due to its uniform particle size distribution and quick water removal (Gouin 2004). Gum Arabica is a widely used coating material for essential oils. It is a suitable choice for powder formulation due to its high solubility, surface activity, low viscosity, good emulsifying ability, non‐toxicity, and tastelessness. The impact of gum Arabic as a wall material on the essential oil's retention following microencapsulation was another major focus of the current investigation. Gum Arabica is a common essential oil coating material. Its greater water solubility, surface activity, low viscosity, good emulsifying ability, non‐toxicity, and tastelessness make it a viable option for powder formulation. There are some research works on encapsulated thyme essential oils. For example, Barrera‐Ruiz et al. (2020) worked on *Thymus capitatus* essential oil encapsulated in chitosan nanoparticles. Jovanović et al. (2021) investigated wild Thyme ( *Thymus serpyllum* L.) EO using freeze vs. spray drying methods employing gelatin as wall material. There are also studies on nano‐encapsulation of thyme essential oil in a chitosan‐Arabic gum system (Xu 2019). In addition, nano‐encapsulation of *T. capitatus* and *Thymus algeriensis* essential oils by using different coating agents was studied by (Jayari et al. 2022).

Therefore, the primary objective of this study was to characterize the microencapsulated *Thymus schimperi* essential oil prepared using both spray and freeze‐drying techniques, gum Arabic serving as the carrier material. This characterization involved a comprehensive evaluation of the physicochemical properties of the resulting microcapsules, including their morphology, particle size, encapsulation efficiency, thermal stability, and moisture content. In addition, this report evaluates and compares spray and freeze‐drying for microencapsulating *T. schimperi* essential oil with gum Arabic. Suitability is assessed by considering encapsulation efficiency, storage stability, bioactivity retention, moisture/surface oil levels, and key physical properties (morphology, density). Furthermore, the study aims to assess the stability of the encapsulated essential oil by using the percentage of DPPH radical scavenging activity. In essential oil, the DPPH assay serves as a sensitive indicator of the *functional stability* of the encapsulated essential oil, specifically concerning its antioxidant properties, which are often among the most vulnerable to degradation during storage. A method that results in an encapsulated product maintaining a high percentage of DPPH inhibition over an extended period is deemed more effective in protecting the oil from oxidative breakdown.

## 2 Materials and Methods

### 2.1 Sample Collection and Preparation

Fresh samples of *T. schimperi* collected from North Shewa, Ankober (kundi) which were found to be 190 km away from Addis Ababa, the capital city of Ethiopia, in the period between December and February 2021/2022. Plant specimens were transported to the laboratory in an ice bag. The species identification was made by a botanist at the Department of Biology, Debre Birhan University.

#### 2.1.1 Sample Preparation

Collected fresh samples were washed to remove dirt and dried at room temperature under the shade. Dried leaf was carefully separated from woody parts and stored in a polyethylene bag at room temperature until used.

#### 2.1.2 *Thymus schimperi* Essential Oil Extraction

Essential oil from dried leaf of the plant (300 g) was extracted by hydrodistillation. The obtained oil was dried using anhydrous sodium sulfate and stored in amber colored glass vials at 4°C until used.

#### 2.1.3 Preparation of Emulsions

Emulsion for microcapsule production was prepared following a previously described method (Krishnan et al. 2005). In brief: solutions with (30%) gum Arabic were prepared by dissolving gum Arabic (Colloides Naturels Brasil, São Paulo, Brazil) in distilled water. For the purpose of ensuring complete saturation of the polymer molecules, the solution was made the day before emulsification and left overnight at room temperature. Using a rotor‐stator blender (EURO‐ST 60 TS 000), enough *T. schimperi* essential oil to provide a mass ratio of 1:3 (v/w) was gradually added to the gum Arabic solution while stirring at 3000 rpm for 10 min. To achieve full emulsification, the suspension was subjected to 30 min of ultrasonication using a Benchmark Pulse 150 Ultrasonic Homogenizer. The feeding liquid for the spray and freeze‐drying procedures was the emulsion. About 800 mL of sample was prepared for each drying and encapsulated powder production procedure.

### 2.2 Microencapsulation by Spray Drying

The solution was subjected to in a Lab Plant MSD/EV 0.5 spray‐dryer with a top spray atomizer. Feeding was performed by a peristaltic pump. During the drying process, the emulsion was stirred and maintained at room temperature. The inlet and outlet temperatures were 150°C and 65°C–70°C, respectively, and the long air pressure was 3 bars. Microcapsules obtained were stored in a brown bottle under refrigeration at a temperature of around 8°C (Burhan et al. 2019).

### 2.3 Microencapsulation by Freeze Drying

The prepared emulsion was subjected to a freeze‐drying (Lablyo Plus Frozen in Time, UK Laboratory) at −55°C, 0.05 mbar for 48 h. The freeze‐dried microcapsules were packed in foil bags and stored in a desiccator for 30 min in order to stabilize temperature. The microcapsules were ground by mortar and pestle. The ground microcapsule was put in amber glass until used for analysis (Wilkowska et al. 2016).

### 2.4 Physical Characterization of the Microcapsules

#### 2.4.1 Moisture Content

Moisture content of the capsules was determined following a reported method (Fernandes, Borges, and Botrel 2013). Weight loss of microcapsules expressed in percentage (%) was determined after oven‐drying at 105°C to a constant weight measured.

#### 2.4.2 Bulk Density (*ρ*_bulk_) and Bulk Tapped Density (*ρ*_tapped_)

After being carefully filled to the 5‐mL mark in a tared graduated cylinder, the microcapsules were weighed. The bulk density (*ρ*_bulk_) was determined using the volume taken straight out of the cylinder using the following formula: mass of powder/bulk volume (Botrel and Oliveira 2014). 0.6 g of powder was freely poured into a 10 mL glass graduated cylinder in order to determine the tapped density. The samples were then manually tapped by lifting and lowering the cylinder under its own weight vertically until there was no discernible volume difference between subsequent measurements. The powder tapped density was calculated as mass of powder/tapped volume (g/mL) given the mass (m) and apparent (tapped) volume (V) of the powder (Botrel and Oliveira 2014).

#### 2.4.3 Flowability and Cohesiveness

According to Fernandes, Victória, et al. (2013) and Fernandes, Borges, and Botrel (2013), the cohesiveness and flowability of the microcapsules were assessed using the Hausner ratio (HR) and Carr index (CI), respectively. According to Equations (1) and (2), CI and HR were computed using the microcapsules' bulk (*ρ*_bulk_) and tapped (*ρ*_tapped_) densities.

(1) $$\mathrm{CI}=\frac{\text{Tapped density}-\text{Bulk density}}{\text{Tapped density}}\times 100$$

(2) $$\mathrm{HR}=\frac{\text{Tapped density}}{\text{Bulk desity}}$$

#### 2.4.4 Solubility of Microcapsules

Solubility of microcapsules was examined following the method described by Jovanović et al. (2021). In brief, 0.2 g of dried microcapsules was dissolved in 20 mL of distilled water and continuously stirred on a magnetic stirrer plate for 30 min at room temperature. Subsequently, the samples were centrifuged (Bench‐Top centrifuge, NF 200) at 4500 rpm for 10 min. The supernatant was dried at 105°C until a constant weight was measured. Based on the obtained and initial mass of the samples, the solubility of microcapsules was calculated and results expressed as a percentage.

#### 2.4.5 Total and Surface Oil Determination

Total oil content of the microcapsules was determined by dissolving 5 g of microcapsules in 150 mL of distilled water followed by distilling the mixture for 2 h using a Clevenger‐type apparatus. Five milliliters of diethyl ether were added to separate the essential oil from the water phase. Diethyl ether oil solution was collected in a pre‐weighed Erlenmeyer flask, and the solvent evaporated at room temperature for 24 h (Beristain et al. 2001). After removal of the solvent, the weight of the Erlenmeyer flask was measured and total oil of microcapsules was calculated by weight difference.

Surface oil of the microcapsules was determined by the method described by Barrera‐Ruiz et al. (2020). Twenty milliliters of hexane was added to 2 g of microcapsules, and the mixture was stirred at 300 rpm for 10 min. The suspension was filtered through Whatmann No1 filter paper, and the residue washed with three portions of 20 mL of hexane. The filtrate was collected in a pre‐weighed round bottom flask. The solvent was removed in vacuo (DW‐ORE‐3000). Finally, the oil encapsulation efficiency (EE) was calculated using Equation (3) (Luo et al. 2019):

(3) $$\mathrm{EE}=\frac{\text{Total oil}-\text{Surface oil}}{\text{Total oil}}\times 100$$

#### 2.4.6 Particle Size Distribution

The laser diffraction light scattering wet method was used to determine the average diameter and particle size distribution using Nanosizer (Malvern Instruments, Malvern3600). A 0.1 g of the microcapsules was suspended in 20 mL distilled water as a dispersing medium and sonicated for 5 min with an ultrasound sonicator. This suspension was used for the average diameter measurement (Liu et al. 2023).

#### 2.4.7 Morphology

Micrographs of spray and freeze dried encapsulated samples were examined using a scanning electron microscopy (SEM) (JCM‐6000Plus), at an acceleration voltage of 15 kV in a magnification range of 900–1200× (Marques et al. 2021).

#### 2.4.8 Thermogravimetric Analysis (TGA)

Thermal properties of the microcapsules were performed by thermogravimetric analysis (TGA) (Beijing, HCT‐1). Measurements were performed under a nitrogen atmosphere of 20 mL/min, with a constant heating rate of 10°C/min, with a temperature ranging from 25°C to 700°C. The thermogravimetric curves obtained were analyzed using Origin Pro 2021 software (Karaaslan et al. 2021).

#### 2.4.9 Fourier Transform Infrared Spectroscopy (FTIR)

Chemical groups and binding arrangement of the constituents present in the samples were determined using a Fourier Transform Infrared Spectrometer (FTIR) (iS50 ABX, Model AUP 1600388) equipped with ATR cell. Analysis was performed in the range from 4000 to 500 cm^−1^, with resolution adjusted to 4 cm^−1^ per sample.

#### 2.4.10 In Vitro Release Kinetics

Release of thyme oil from the microcapsules was determined following a reported method (Mehran et al. 2020). Briefly, release profiles of thyme oil from microcapsules were determined by suspending 0.5 g of microcapsules in 25 mL of ethanol: water (1:1) mixture. The suspension was stirred at 100 rpm, at 25°C for 5 min. At predetermined times, aliquots of the supernatant were removed from the release media and replaced with an equal volume of the ethanol: water mixture to maintain a constant volume. The supernatant was analyzed in a spectrophotometer at 280 nm, and the cumulative release percentage was plotted as a function of incubation time. The procedure was realized in triplicate. All samples' absorbance was measured in a UV‐spectrophotometer (model: L1‐295, Bangladesh) and correlated with thyme essential oil calibration curves (*λ* = 280 nm) with the concentration (5–30 μL). The concentrations and the percentage of thyme oil released over time were calculated from the calibration curve.

#### 2.4.11 Storage Stability Studies

The storage stability of the microcapsules was performed using accelerated stability testing methods. The microcapsules were stored in a laboratory electric oven at 40°C for 15 days, where DPPH inhibition capacity was performed within 3‐day intervals (Suhag and Nanda 2017).

### 2.5 Degradation Kinetic Study of Microcapsules and Shelf‐Life Estimation

#### 2.5.1 Kinetic Reactions

The kinetic reaction strategy is the most straightforward method for testing shelf lives. The kinetic data are used to assess how the deterioration process acts as a function of time in order to forecast the shelf life. It is the idea that food product quality may be measured using a shift in response (Phimolsiripol and Suppakul 2016). The kinetic equation is expressed as;

(4) $$\text{Rate}=\left(-\mathrm{dc}\right)/\mathrm{dt}=K{\left[A\right]}^{n}$$

$$\left(-\mathrm{dc}\right)/\mathrm{dt}=K$$

dc=Kdt

where, *dc* is the change in % DPPH radical scavenging with respect to change in time *t*, *n* indicates order of reaction; minus sign indicates that the activity is decreasing.

Most of food qualities loss are represented by zero or first‐order reactions (Suhag and Nanda 2017). The degradation of microcapsules made by spray and freeze‐drying techniques was calculated by using the standard equation for a first‐order kinetic model as follows:

(5) $$\ln C=\ln C_{o}-\mathrm{Kt}$$

where *C* is the DPPH percent of inhibition at time *t*; *C*_o_ is the percent of DPPH inhibition at time zero; *K*, the degradation rate constant (day^−1^) is obtained from the slope of a plot of the natural log of *C*/*C*_o_ versus time; and *t*, being the storage time (days). The shelf‐life corresponds to the time at which the microcapsules lose their activity with respect to zero time of storage and it was calculated by the equation:

(6) $$S_{t}=\left(\ln C_{o}-\ln C\right)/k$$

#### 2.5.2 Statistical Data Analysis

A comparative analysis of mean values between the spray‐dried and freeze‐dried microcapsules was conducted using one‐way analysis of variance (ANOVA). Statistical differences were considered significant at *p* < 0.05. Data analysis was performed using SPSS software (version 26.0). Graphs were created using Origin Pro software (version 2021) to depict the results.

## 3 Result and Discussion

### 3.1 Physical and Chemical Properties of Microcapsule

Table 1 presents a comparative analysis of two drying methods, Spray drying and Freeze drying, based on various physical and chemical properties of the resulting product. The parameters evaluated include moisture content, solubility index, bulk density, tapped density, compressibility index, Hausner ratio, total oil content, surface oil content, and encapsulation efficiency. Each value is presented as a mean ± standard deviation, with letters (a, b) indicating potential statistical differences between the two drying methods for each parameter.

**TABLE 1:** Physical characteristics of freeze and spray dried microcapsules.

Drying T.	M. content (%)	*S. index* (%)	B. density (g/mL)	T. Density (g/mL)	C. index (%)	H. ratio	T. oil (mg/g)	S. oil (mg/g)	E.E. (%)
Spray	2.8 ± 0.1^b^	92.00 ± 0.6^a^	0.23 ± 0.00^b^	0.5 ± 0.03^a^	53.3 ± 3.00^a^	2.13 ± 0.2^a^	2.6 ± 0.00^b^	1.1 ± 0.00^b^	71.2 ± 0.3^a^
Freeze	4.7 ± 0.2^a^	57.8 ± 2.6^b^	0.28 ± 0.00^a^	0.4 ± 0.02^b^	30.27 ± 6.65^a^	1.44 ± 0.14^b^	4.7 ± 0.00^a^	1.5 ± 0.00^a^	59.52 ± 0.95^b^

Abbreviations: B. density, bulk density; C. index, carr index; E.E., encapsulation efficiency; H. ratio, hosner ratio; M. content, moisture content; S. index, solubility index; S. oil, surface oil; T. density, tapped density; T. oil, total oil. Letters (a, b) indicating potential statistical differences between the two drying methods for each parameter.

#### 3.1.1 Moisture Content (M. Content [%])

Spray drying resulted in a lower moisture content (2.8% ± 0.1%) compared to Freeze drying (4.7% ± 0.2%). The letters “b” and “a” associated with Spray and Freeze drying, respectively suggest that this difference is statistically significant. Lower moisture content is often desirable for product stability and shelf life. Water functions as a plasticizer, lowering the glass transition temperature, which can lead to capsules becoming sticky and forming lumps, thereby decreasing molecular mobility (Böger et al. 2018). During spray‐drying, due to the exposure to a larger surface area, heat and mass transfer rate increase, leading to lower moisture contents (Cassol et al. 2021). The high moisture content in freeze‐dried powders may result from rapid freezing at low temperatures, where formed pores can act as barriers to sublimation, thereby limiting mass transfer and leading to increased moisture retention (Pudziuvelyte et al. 2020). The moisture content of the spray dried oil in the current study was 2.8% close to (0.26%–3.16%) that reported by Fernandes, Victória, et al. (2013) and Fernandes, Borges, and Botrel (2013) at temperature of (135°C–195°C), (10%–30%) gum and (0.5–1 L/h) feeding rate. In general, low moisture content indicates good quality of microcapsules including storage stability and preventing degradation by either oxidation and/or hydrolysis.

### 3.2 Bulk Density (B. Density [g/mL]) and Tapped Density (T. Density [g/mL])

Freeze drying resulted in a higher bulk density (0.28 ± 0.0 g/mL) than Spray drying (0.23 ± 0.0 g/mL). The letters “a” and “b” suggest this difference is statistically significant. Bulk density relates to how much powder can fit into a given volume and can affect packaging and transportation costs (Badee et al. 2012). Although the tapped density values were reported as 0.5 ± 0.02 g/mL for Spray drying and 0.4 ± 0.03 g/mL for Freeze drying. The letter ‘a’ for both methods might indicate no statistically significant difference despite the mean values, potentially due to the large variance in the spray‐dried sample. Tapped density is measured after vibrating the powder and is generally higher than bulk density. The tapped density of spray dried encapsulated *T. schimperi* EO has higher value than that of Freeze‐dried *T. schimperi* EO. From this low bulk density of the spray‐dried powder has a relation with porosity, meaning it contains more air spaces between particles compared to the freeze‐dried powder. But high tapped density powders that compact well typically flow better, which is important in many industrial processes such as filling capsules or mixing. Similar results were reported by the encapsulation of rosemary essential oil by spray (0.20–0.26 g/mL) for bulk density and 0.29 and 0.41 g/mL for tapped density (Botrel and Oliveira 2014). Another study by Marques et al. (2021) on spray‐dried thyme essential oil reported a value in the range of 0.20 ± 0.01–0.33 ± 0.02 g/mL for bulk density and 0.33 ± 0.02–0.39 ± 0.02 g/mL for tapped density with different blend ratio of whey protein isolate, maltodextrin and chitosan as a coating agent.

Freeze drying microcapsules showed relatively higher bulk density and lower tapped density than that of spray dried ones. This variation could typically be due to the spray drying encapsulation technique, which produces smaller and more spherical particles that do not pack tightly in their initial state, resulting in lower bulk density. However, when tapped, these spherical particles can rearrange and pack more efficiently, leading to a higher tapped density. In contrast, freeze drying often results in higher bulk density, which may be due to more irregular particles with more structure. These particles might not compact as efficiently when tapped, leading to a smaller difference between bulk and tapped densities. On the other hand, higher tapped density indicates that spray‐dried encapsulated *T. schimperi* EO is easier to handle, transport, and package, especially in automated processes. In addition, the ability to compact tightly means that spray‐dried encapsulated *T. schimperi* EO may occupy less volume when packaged, potentially reducing packaging size and costs. Small packing volume leads to packaging feasibility, easier transport, and an overall increase in economic value (Burhan et al. 2019).

#### 3.2.1 Flowability and Cohesiveness

Carr index and Hausner ratios of microcapsules are the two quality control parameters which indicate the flow properties of the powders (Afonso et al. 2019). The Carr index and Hausner ratio of spray and freeze‐dried encapsulated *T. schimperi* essential oil are presented in Table 1.

##### 3.2.1.1 Carr Index (C. Index [%])

Spray drying resulted in a higher compressibility index (53.3% ± 3.0%) compared to Freeze drying (30.28% ± 6.7%). The compressibility index is an indicator of a powder's flowability, with a higher index suggesting poorer flowability. The letter “a” for both might suggest no statistically significant difference despite the difference in means, possibly due to the standard deviations.

##### 3.2.1.2 Hausner Ratio (H. Ratio)

Consistent with the compressibility index, Spray drying yielded a higher Hausner ratio (2.13 ± 0.2) than Freeze drying (1.44 ± 0.14). The Hausner ratio is also a measure of powder flowability, with a higher ratio indicating poorer flow. The different letters (“a” and “b”) suggest a statistically significant difference, indicating that the freeze‐dried powder has better flow properties than the spray‐dried powder.

The higher Hausner Ratio and Carr index reinforce the idea that spray and freeze‐dried encapsulated *T. schimperi* EO has poorer flowability, which could lead to issues like clogging in uneven filling in containers or difficulties in mixing with other powders. It can be concluded that both encapsulation techniques give microcapsules with bad flow properties. In the study of Fernandes, Victória, et al. (2013) and Fernandes, Borges, and Botrel (2013) on rosemary essential oil, values of CI (23.09%–40.22%) were reported for the powders produced by spray drying. In another study by Fuchs et al. (2006) on vegetable oil, a value of CI 44% was found for the powders produced by spray drying. Jinapong et al. (2008) obtained values varying from 32% to 40% for atomized soy milk.

Several factors can contribute to a high Carr index in microcapsules. For example, small particle size tends to have a larger surface area relative to their volume, leading to increased interparticle forces that lead to flow resistance (Fernandes, Victória, et al. 2013; Fernandes, Borges, and Botrel 2013). The chemical profile of the microcapsule surface plays a vital role in this property because flowability encompasses overcoming the surface interactions among the particles (Fernandes, Victória, et al. 2013; Fernandes, Borges, and Botrel 2013). A higher Hausner ratio indicates that the microcapsules are more cohesive and less capable of flowing freely. The results of the Hausner ratio obtained in this study were 2.13 ± 0.13 and 1.44 ± 0.12 for spray and freeze‐dried powders, respectively. Thus, the microcapsules produced by both drying techniques can be classified as one with poor flow properties. In general, the flow property of microcapsules is affected by moisture content, particle size, and surface composition (Suhag et al. 2024).

##### 3.2.1.3 Solubility Index (S. Index [%])

Spray drying yielded a significantly higher solubility index (92.00% ± 0.6%) than freeze drying (57.8% ± 2.6%). The “a” for spray and “b” for freeze indicate a statistically significant difference. A higher solubility index suggests that the product dissolves more readily in a water, which is a crucial quality parameter for many powdered products. High solubility means that the microcapsules can effectively disperse and integrate into the solvent without forming clumps or leaving residues. This property is crucial for applications in the food industry, where the microcapsules need to dissolve completely into food matrices to ensure uniform distribution of the encapsulated ingredients (Marques et al. 2021). Solubility of the *T. schimperi* EO microcapsules prepared by spray and freeze drying encapsulated was observed on is presented in Table 1. According to the data, the spray drying encapsulation technique afforded powder that is more soluble than the one prepared by freeze drying technique. The high solubility of powders produced by spray drying technique could be due to the fact that the smaller the particle size, the greater the surface area available for hydration. The powder solubility from spray dried microcapsule of *T. schimperi* EO for the current study was close to (92.06%) that reported by Rigon and Zapata Noreña (2016) on spray drying of bioactive compounds from black berry (88.2%–97.4%). In general, solubility of microcapsules is affected by factors like the choice of encapsulating agent, hydrophilicity/hydrophobic nature of coating agent, the properties of core material, drying method, particle size, and moisture content of the microcapsules. In the current study, drying methods, particle size, and moisture content may have a crucial effect in the variation on solubility of spray dried microcapsule from freeze dried one.

#### 3.2.2 Total Oil (T. Oil [mg/g]), Surface Oil (S. Oil [mg/g]) and Encapsulation Efficiency (E.E. [%])

Surface oil content is the oil observed on the outer surface of the microcapsule wall. Existence of oil on the outer surface is undesirable as it can be damaged while coming in contact with the surrounding environment. Percentage surface oil is calculated by comparing the oil that is found on the surface with the oil that is purposely added to suspension to from the total oil content. As presented in Table 1 Freeze drying retained a significantly higher amount of total oil (4.7 ± 0.00 mg/g) compared to Spray drying (2.6 ± 0.00 mg/g). The letters “a” and “b” confirm a statistically significant difference. This suggests that the freeze‐drying process is more effective at preserving the total oil content in the product. Similar to total oil, Freeze drying resulted in slightly more surface oil (1.5 ± 0.00 mg/g) than Spray drying (1.1 ± 0.0 mg/g). The letters “a” and “b” indicate a statistically significant difference. Although the absolute amounts of surface oil are low for both, the difference is statistically notable. Surface oil can affect the product's stability and tendency to clump. Lower surface oil on microcapsule indicates better encapsulation efficiency (Yaman et al. 2023). Thus, in spray drying, the liquid containing the encapsulated material is atomized into fine droplets and then rapidly dried using hot air. This process creates a fine powder with a relatively uniform and consistent particle size. The rapid drying helps in forming a solid shell around the oil or active ingredient, which effectively encapsulates it and minimizes the amount of surface oil. In contrast, freeze drying involves freezing the material first and then sublimating the ice under a vacuum. This process is slower and results in a more porous and irregular structure. Formation of ice crystals during freezing can create larger voids in the product. When the ice sublimes, it leaves behind a structure that may not fully encapsulate the oil, leading to a higher likelihood of oil remaining on the surface.

Spray drying showed a significantly higher encapsulation efficiency (71.2% ± 0.3%) than Freeze drying (59.52% ± 0.95%). The letters “a” and “b” confirm a statistically significant difference. Encapsulation efficiency is a measure of how much of the active compound (likely the oil in this case) is successfully entrapped within the powder particles, protecting it from degradation (Table 1). Thus, encapsulation efficiency of freeze‐drying technique is considered to be lower due to the higher surface oil compared to that of spray dried microcapsule. This phenomenon was also noted by others researchers (Ogrodowska et al. 2020). This is mainly due to the difference in the dehydration mechanism in the spray and freeze‐drying method. Silva et al. (2016) noted that ice crystal formation during the pre‐encapsulation freezing stage causes emulsion droplets to rupture, facilitating the release of oil from the particles.

Compared to previous studies, Tomazelli Júnior et al. (2018) on encapsulation of thyme essential oil by spray drying with maltodextrin as wall material and casein sodium salt as emulsifier, encapsulation efficiency of the produced powder was found to be 87.16%. In accordance with Tomazelli Júnior et al. (2018) our result of the current study has lower encapsulation efficiency (71.2%) for spray drying using gum Arabic as wall material. This variation may be due to the variation of wall materials. In general, the encapsulation efficiency of a microcapsule is influenced by multiple factors including the type of wall materials, the concentration of core material in a suspension, encapsulation techniques and parameters (inlet out let temperature) and the method used to determine encapsulation efficiency, etc. (Luo et al. 2019). In conclusion, the choice between spray drying and freeze drying depends on the desired properties of the final product. Spray drying appears to be more suitable when high solubility and encapsulation efficiency are paramount, despite leading to lower oil retention and poorer flowability. Freeze drying, on the other hand, is more effective at preserving oil content and results in better powder flow properties, although it yields a product with higher moisture content and lower solubility and encapsulation efficiency. The statistical indicators (letters a and b) highlight that many of these differences are statistically significant, reinforcing the distinct outcomes of the two drying methods on the product characteristics.

#### 3.2.3 Morphology of the Microcapsules

**FIGURE 1:**
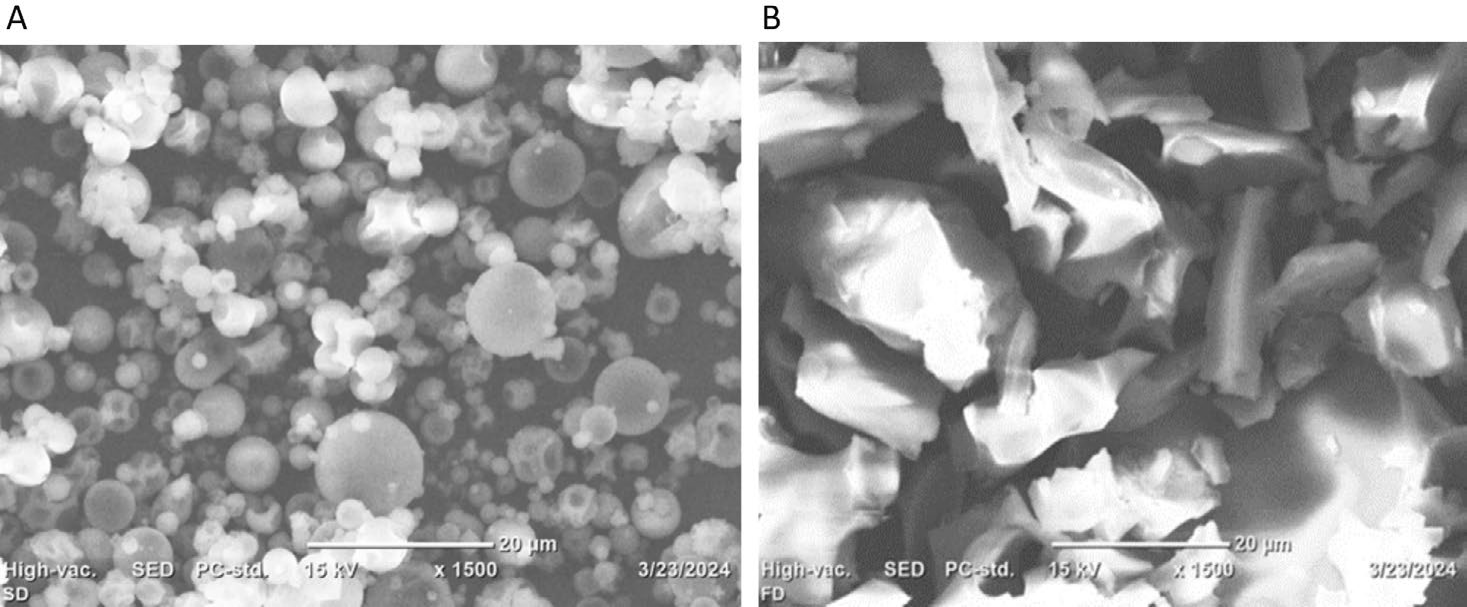
Scanning electron microscopic photographs of microcapsules (A) for spray and (B) for freeze dried powders.

Property of microcapsules is related to its morphology (Tai et al. 2023). The scanning electron microscopic result from the current study is shown in Figure 1. There was a structural difference between spray‐and freeze‐dried encapsulated *T. schimperi* EO. For the spray‐dried sample (Figure 1A), most of the particles showed a spherical, smooth, and regular shape. Their outer surfaces are characterized by the presence of shallow dents or faint traces of shrinkage. These dents were due to contraction and shrinkage of liquid droplets during the early stage of drying and cooling, resulting in surface indentations (Chen et al. 2013; Mehran et al. 2020).

Unlike the spray‐dried samples, the structure of the freeze‐dried microcapsules showed irregular, sharp, and broken glasslike smooth surfaces, reflecting the result of grinding after drying (Figure 1B). The irregular, sharp, and broken glasslike structures with smooth surfaces in freeze‐dried microcapsules are primarily due to the rapid freezing and sublimation processes, and potential mechanical stress during or after the freeze‐drying process. The same observations were reported (Jovanović et al. 2021) for freeze *and* spray drying for dry wild thyme extract using gelatin as a coating material. In general, microcapsules produced by spray drying have a characteristic spherical small size, smooth surfaces, and no features of hollowness, indicating that the microcapsule had good rigidity, which could protect the thyme essential oil from the influence of air, light, and high temperature.

#### 3.2.4 Particle Size of Microcapsules

**FIGURE 2:**
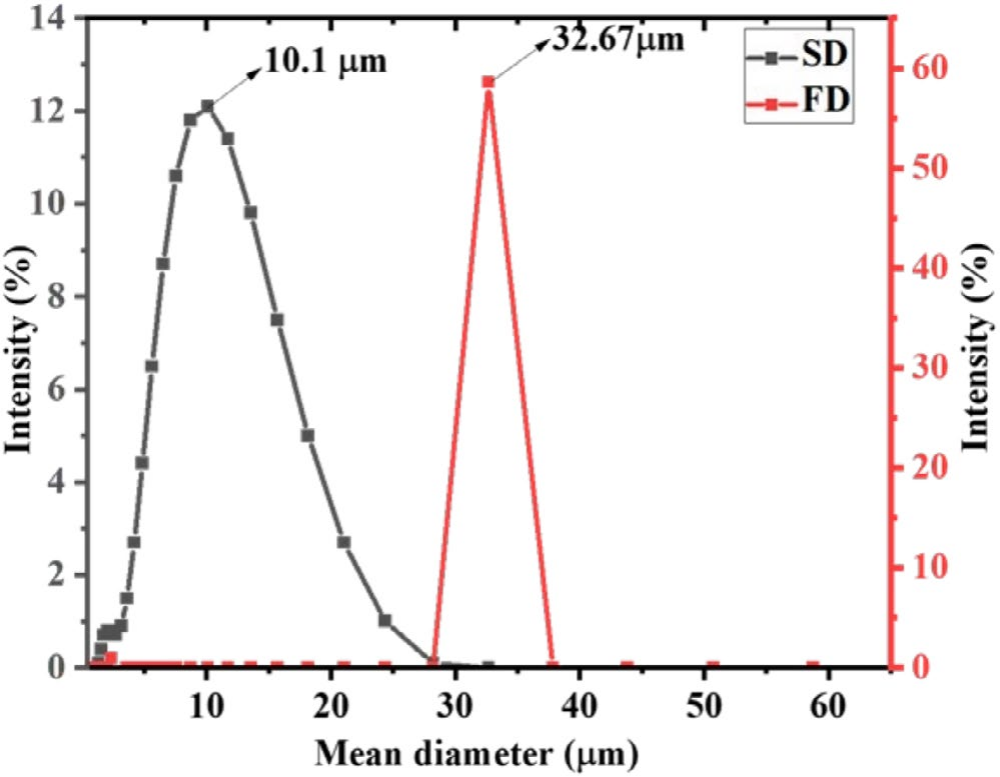
Particle size distribution of spray and freeze‐dried microcapsules.

The particle size of the microcapsules of thymus essential oil was measured using Zetasizer (Malvern Instruments, Malvern3600) (Figure 2). Values obtained for spray dried powder ranged from 1.3 to 24.4 μm, with an average size of 10.1 μm. The freeze‐dried powder had particles in the range of 28.21–37.84 μm with an average size of 32.67 μm. This variation may be due to the involvement of atomizing a liquid sample into fine droplets that are rapidly dried by hot air in a spray dryer. The rapid evaporation of solvent (often water) causes the droplets to solidify quickly, forming smaller, more uniform particles. The fast‐drying process leads to the formation of smaller particles with a relatively consistent size. In contrast, freeze drying, with its slower sublimation process and formation of porous structures, tends to produce larger and less uniform microcapsules. The diameter of the microcapsules produced depended on the material properties, concentration, viscosity of the encapsulating material, and the operating conditions of the spray dryer (Jafari et al. 2008). The particle size range for the spray dried microcapsules was relatively narrow, indicating that the microcapsules were of uniform size, which was consistent with available results in the literature (Liu et al. 2023).

The small particle size of microcapsules indicates higher encapsulation efficiency because the smaller particle size allows for a more uniform distribution of the core material within the wall matrix. The increased surface area of smaller particles can improve interactions between the core and the wall material. Similar particle size values were reported in the literature with a size of 10.37 ± 0.0184 μm (Tomazelli Júnior et al. 2018) for encapsulating thyme oil by spray drying using oil‐inin‐water (1:4) emulsion. Similar particle sizes were observed in oregano essential oil microencapsulation (7.5–18.6 mm) using a mixture of maltodextrin (MD), gum Arabic (GA), and modified starch as a coating agent (Alvarenga Botrel et al. 2012). In general, particle size and shape of the microcapsules greatly influence appearance, flowability, and powder dispersibility (Alvarenga Botrel et al. 2012). As a conclusion, the particle size of microcapsules is influenced by a combination of factors related to the encapsulation method, process conditions, properties of the feed solution, wall and core materials, mixing and agitation conditions, environmental factors, and additives.

##### 3.2.4.1 FTIR Analysis

**FIGURE 3:**
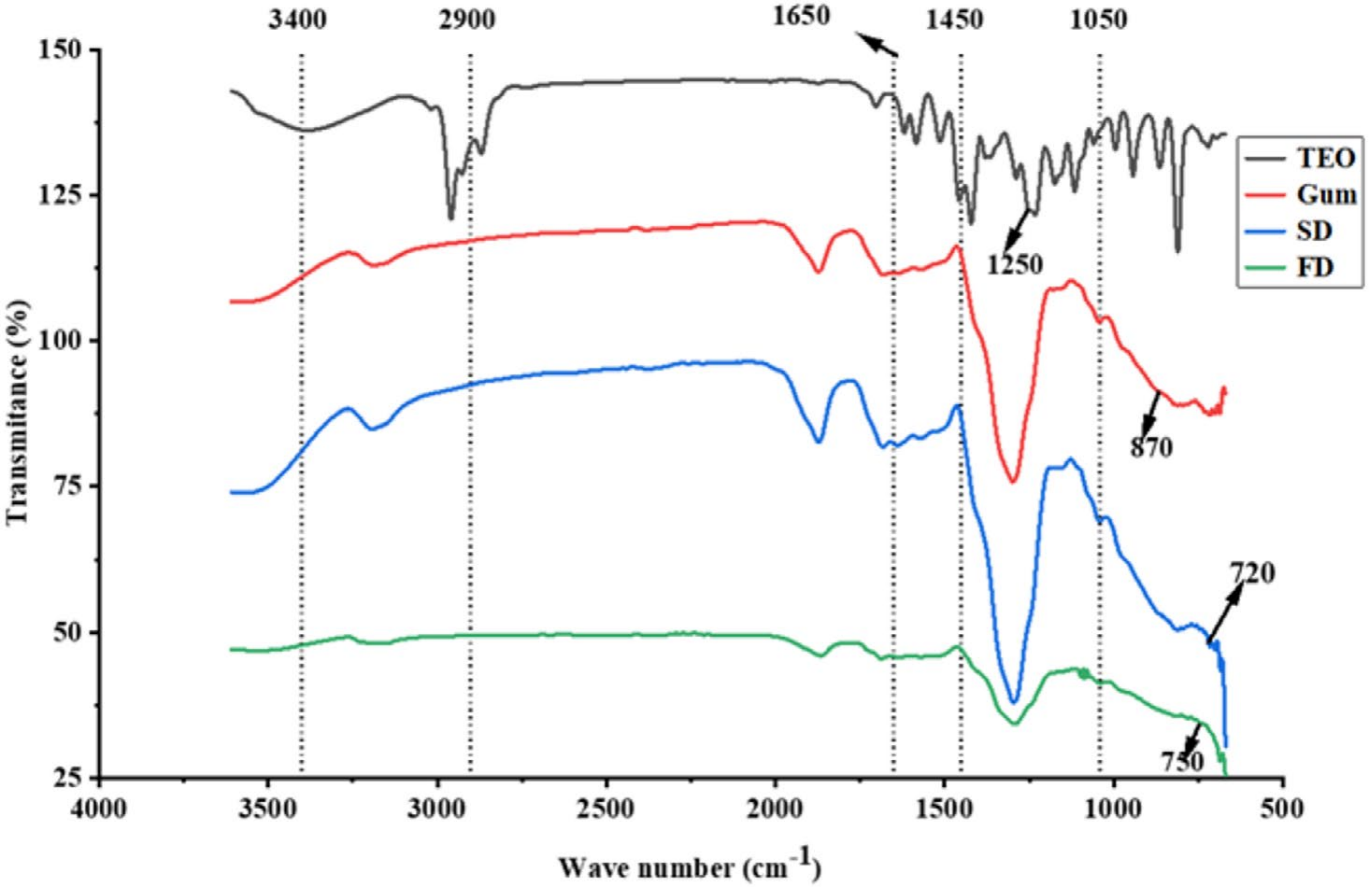
FTIR spectrum. FD, freeze‐dried microcapsules; Gum, Gum Arabic; SD, spray‐dried; TEO, thyme essential oil.

FTIR analysis was performed to evaluate the influence of microencapsulation methods (spray drying and freeze drying) on the chemical constituents of *T. schimperi* essential oil (TEO) using gum Arabic as the wall material. The results of FTIR spectra of TEO, pure gum Arabic, and both freeze‐dried (FD) and spray‐dried (SD) microcapsules are presented in Figure 3.

The TEO sample exhibited a distinct peak around 1250 cm^−1^, which is not prominent in the gum Arabic, SD, or FD spectra. The specific intensity variations observed in Figure 3 at 1250 cm^−1^ for SD and FD relative to gum would depend on the concentration of the encapsulated oil and the specific functional groups within that oil that absorb in this region, as well as any subtle changes induced in the gum Arabic matrix by the different drying methods. Conversely, gum Arabic showed a unique peak at 870 cm^−1^, which was not prominent in the TEO, SD, or FD spectra. This peak is typically associated with C–H bending vibrations or other specific molecular vibrations characteristic of certain polysaccharides or other compounds present in gum Arabic.

The SD sample displayed a distinct peak at 720 cm^−1^, which was not prominent in the TEO, gum Arabic, or FD spectra. This peak may be associated with CH_2_ rocking vibrations, typically found in long‐chain hydrocarbons, and likely results from the interaction of the oil with the wall material (gum Arabic) at high drying temperatures. Similarly, the FD sample exhibited a unique peak around 750 cm^−1^, not present in the TEO, gum Arabic, or SD spectra. This peak is typically associated with C–H bending vibrations, often resulting from the interaction of aromatic compounds with the wall material during low‐temperature drying.

Regarding the observed variations in intensity in this region (3300–3500 cm^−1^) in Figure 3, the intensity of this –OH stretching band is typically expected to decrease and potentially broaden or shift slightly compared to pure gum Arabic. This reduction in intensity is attributed to several factors related to the encapsulation process, like matrix formation, interaction with oil, and lower moisture content. Peaks around 2900 cm^−1^, characteristic of C–H stretching vibrations, suggested the presence of aliphatic hydrocarbons. The peak at 1650 cm^−1^, associated with C=C stretching vibrations, indicated the presence of alkenes or aromatic compounds. A peak at 1450 cm^−1^ corresponded to C–H bending vibrations, commonly found in aliphatic compounds. Additionally, a peak at 1050 cm^−1^, due to C–O stretching vibrations, was observed, typically found in alcohols, ethers, or esters. In summary, the FTIR analysis revealed specific peaks associated with the encapsulation methods and the interactions between the essential oil and wall materials. These findings highlight chemical variations introduced by freeze‐drying and spray‐drying techniques, providing perceptions into the structural composition and chemical integrity of the essential oils with gum Arabic.

#### 3.2.5 Thermal Properties of Microcapsules

**FIGURE 4:**
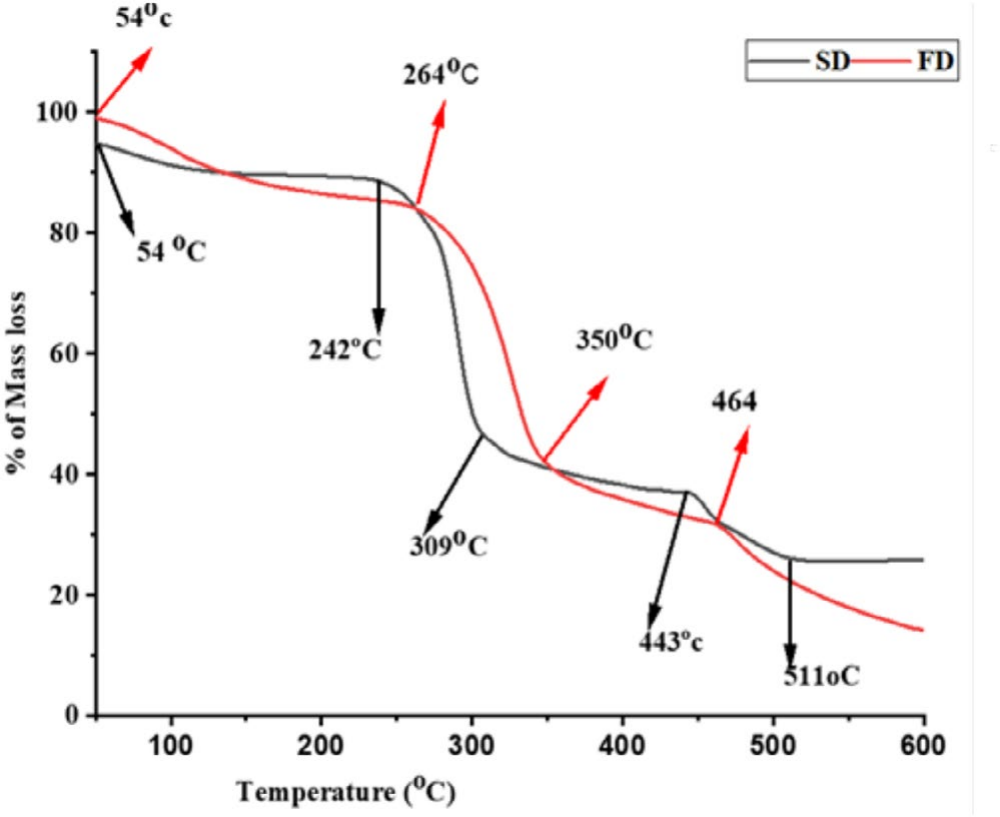
TGA curve of spray‐dried and freeze‐dried encapsulated *Thymus schimperi* essential oil.

The result from thermogravimetric analysis (TGA) of spray dried and freeze‐dried microencapsulated *T. schimperi* essential oil is presented in Figure 4. The result shows the presence of four stages of mass loss. The first stage of mass loss was observed at a temperature of 54°C–242°C for spray dried and 54°C–281°C for freeze dried encapsulated EO. The amount of mass loss at this stage was found to be 11.11% and 15.57% for SD and FD encapsulated *T. schimperi* EO, respectively. In this temperature range, removal of water molecules and surface oil was achieved for both samples (spray dried and freeze‐dried microcapsules of *T. schimperi* EO) prepared using gum Arabic as a coating agent.

The second phase of mass loss ranging between 242°C–309°C for SD and 264°C–350°C for FD was due to the thermal degradation of polymer chains of gum Arabic. In this stage, a percentage mass loss of 45.95% and 41.58% for SD and FD, respectively, was observed. The third stage of degradation ranged between 309°C–443°C for SD and 350°C–464°C for FD due to the evaporation of core materials (*T. schimperi* EO). A mass loss of 5.82% and 10.31% was observed for SD and FD microcapsules, respectively. The fourth degradation stage ranged between 464°C–581°C for SD and 489°C–620°C for FD encapsulated *T. schimperi* EO, which may be related to the carbonization of polymeric materials.

The results from the thermogravimetric analysis showed that capsules prepared by freeze drying encapsulation technique had higher mass loss at the first and the third stages than that of spray dried microcapsules. This could be due to the fact that freeze dried microcapsules have higher moisture and surface oil content than spray dried ones. This leads to a higher percentage of mass loss during the first stage thermal analysis. Freeze drying encapsulation technique maintains a high amount of oil in the capsules than spray drying techniques due to the low processing temperature. After degradation of wall material (gum Arabic), the high amount of oil maintained gets evaporated, and this could be the cause for the higher mass loss observed in the third stage in freeze dried microcapsules than that of spray drying. The superior thermal properties of microcapsules from spray and freeze dried techniques are highly beneficial for food applications typical for thermal food processing (baking). Yavari Maroufi et al. (2021) described the three main stages of weight loss for incorporation of thyme essential oil into poly lactic acid (PLA)/guar gum as a wall material. According to their report, the first stage of weight loss was associated with dehydration of water molecules, the second with thermal degradation of polymer chains, and the third with carbonization of polymeric materials. In summary, the thermal properties of microcapsules are affected by factors such as the type and molecular weight of the wall material, nature and volatility of the core material, the encapsulation method, particle size and structure, moisture content, processing conditions, additives, and interactions between core and wall materials.

##### 3.2.5.1 Oil Releasing Property of Microcapsules

**FIGURE 5:**
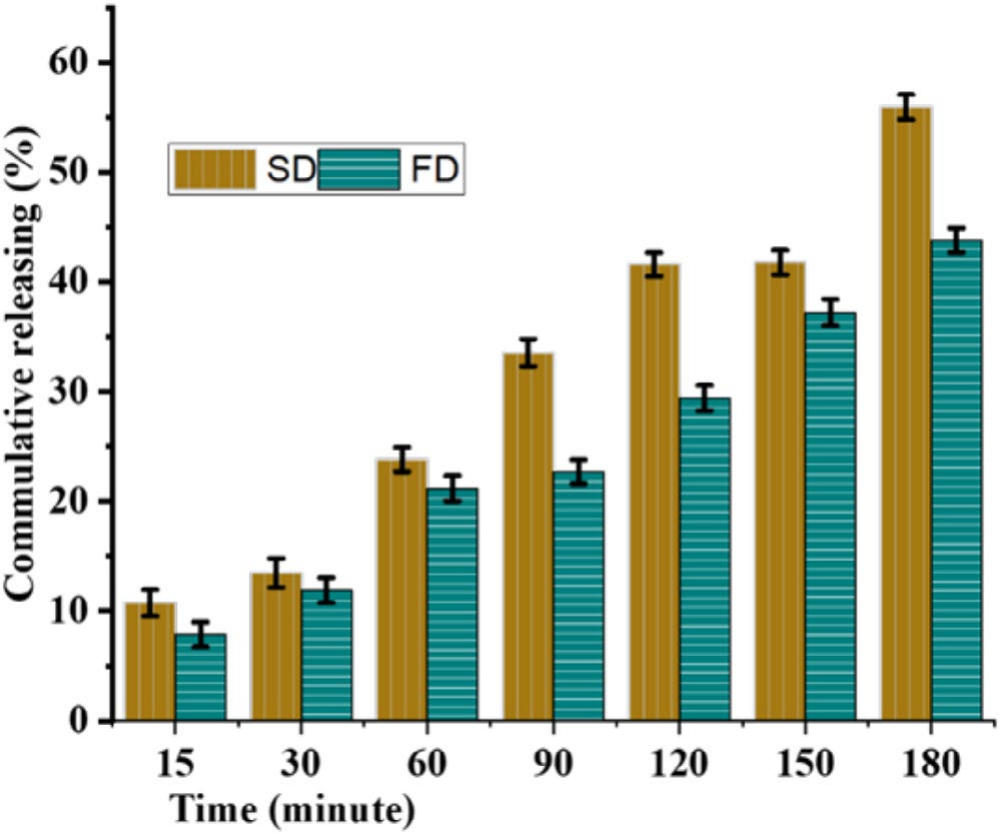
Cumulative oil release graph of spray and freeze‐dried *Thymus schimperi* essential oil microcapsule.

One of the purposes of essential oil encapsulation is to have products with a prolonged release of active components. Thus, the release of *T. schimperi* essential oil from freeze‐dried and spray‐dried gum Arabic encapsulates was evaluated using the 1:1 water–ethanol mixture food simulant (Mehran et al. 2020). The kinetics of oil release was determined at room temperature. The results of in vitro thyme release from microcapsules of spray‐dried and freeze‐dried microcapsules are shown in Figure 5. The results are expressed as the dependence of *C*_*t*_/*C*_*t*o_ on time, where *C*_*t*_ is the essential oil concentration at the time of measurement and *C*_*t*o_ is the amount of oil in the microcapsule. The value of oil released from thyme oil microcapsules prepared by spray and freeze‐drying was measured at specific time intervals using the standard curve of thyme oil at 280 nm. The releasing mechanism of the active component from the microcapsule was in a sustained release form, ensuring that the essential oil is gradually released over time, maintaining its effectiveness for a longer period. This is particularly useful in applications where continuous action is required, such as in food preservation. Spray‐dried microcapsules release a higher amount of EO at a time than freeze‐dried ones. This is probably due to the small particle size of the SD microcapsules that have a high surface area of contact with solvent, thereby increasing the amount of oil released (Jovanović et al. 2021).

To assess the mechanism of thyme oil release from the microcapsules, the data obtained from the release profile best fits Korsmeyer‐Peppas model based on its correlation coefficient (*R* ^2^). The correlation coefficients (*R* ^2^) of SD and FD capsules were 0.992 & 0.996, respectively on Korsemeyer‐Peppas model. Zero order and Higuchi releasing kinetic model the other best fit model next to Korsmeyer‐Peppas as shown in Table 2. If *n*′ ≤ 0.43, diffusion controls release; otherwise, release mechanisms in microcapsules are explained as Fickian diffusion (case I transport), according to the *n*′ values (Table 2). A diffusion and swelling release mechanism (non‐Fickian or anomalous transport) is shown if 0.43 < *n*′ < 0.85. Finally if *n*′ ≥ 0.85 corresponds to zero‐order release kinetics (case II transport) so release depends on swelling of the polymer (Ozdemir et al. 2021). Diffusional exponent values in this work were 0.71 and 0.75 for SD and FD, respectively, suggesting that the release rate is dependent on both the swelling and diffusion processes associated with a non‐Fickian transport mechanism at the same time. The finding from the present study is consistent with that found by Tai et al. (2023) for microcapsule loaded with *Perilla* essential oil for its application in preservation of peaches. In general there are different factors that may influence the diffusion process of microcapsules, such as the interactions between the core and coating materials, the size of the micro particles, and the homogeneity of the essential oils distributed in the micro pores, as well as the consistency of the particle dimensions over time (Luo et al. 2019). Similar observation was made in the study of Herculano et al. (2015) with the best correlation coefficients for Korsemeyer‐Peppas and Higuchi models following the zero order model.

**TABLE 2:** Correlation coefficient values and rate constants of releasing oil from microcapsules using different releasing models.

Capsules	Zero order *R* ^2^	Ko	1st order *R* ^2^	K1	Higuchi *R* ^2^	KH	Korsemeyer Peppas *R* ^2^		*n*
SD	0.97	0.28	0.66	0.007	0.96	4.05	0.992		0.71
FD	0.98	0.22	0.68	0.006	0.961	3.2	0.996		0.75

Abbreviations: FD; freeze drier; SD, spray drier. n: the release mechanism.

##### 3.2.5.2 Storage Stability of Microcapsules

**FIGURE 6:**
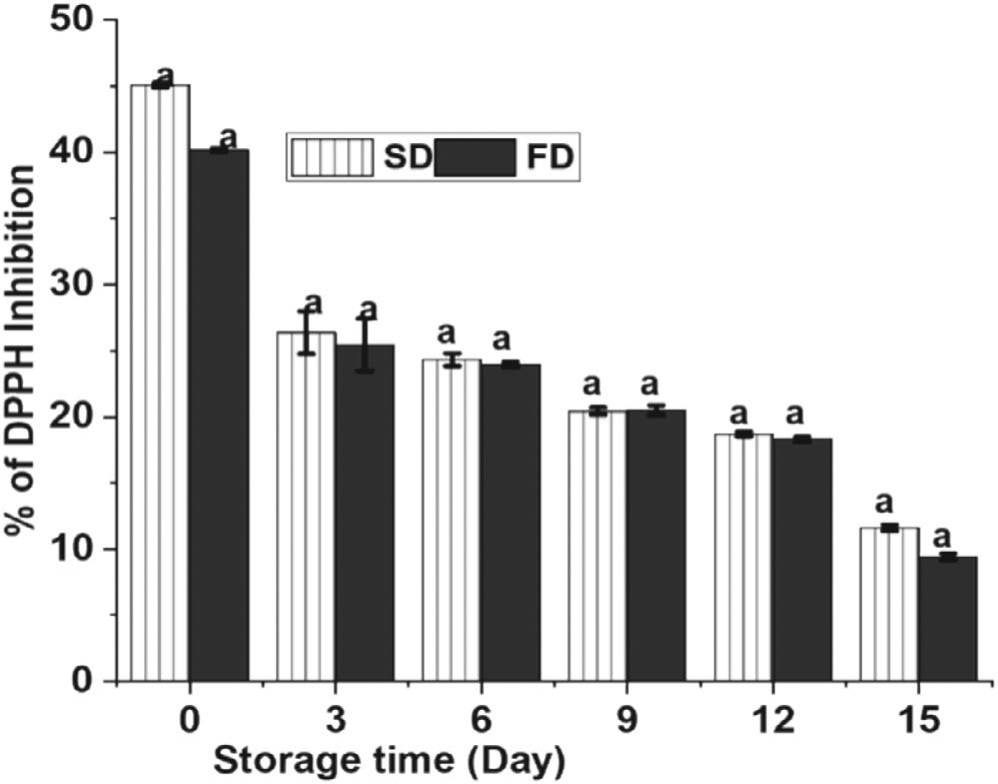
Storage stability of spray and freeze‐dried powder.

Evaluation of storage stability is crucial for determining the effectiveness of a formulation and encapsulation techniques. This is especially critical in the case where the active ingredient is an essential oil known for its instability and volatility. A stable formulation of essential oil should preserve the oil loaded within its solid matrix, reducing susceptibility to chemical degradation and preventing loss through volatilization. Storage stability was assessed by examining the amount of *T. schimperi* oil retained in the gum Arabic matrix, measured through the percentage of DPPH scavenging activity after storing samples for 18 days at 40°C in an oven. In essential oil, the DPPH inhibition assay provides a quantitative measure of the remaining free radical scavenging capacity of the encapsulated essential oil. Since oxidative degradation is a primary concern for essential oil stability during storage, and antioxidant activity is directly counteracted by oxidation, monitoring the change in % DPPH inhibition is a relevant and practical method to evaluate the effectiveness of encapsulation in preserving the essential oil's quality and stability over time. As depicted in Figure 6, the DPPH radical scavenging activity of spray‐dried (SD) and freeze‐dried (FD) microcapsules gradually decreased with increasing storage time. The result obtained indicated that while SD microcapsules demonstrated numerically higher mean DPPH scavenging activity statistical analysis revealed no significant difference in activity compared to FD microcapsules throughout the storage period. Specifically, after 3 days of storage, the DPPH scavenging activity of FD microcapsules was lower than that of SD microcapsules. This suggests that freeze‐drying may result in larger particle sizes, higher moisture content, and lower encapsulation efficiency compared to spray drying, leading to greater loss of active components during storage at 40°C. This observation aligns with findings from previous studies (Chen et al. 2013).

#### 3.2.6 Degradation Kinetics of Microcapsules

**FIGURE 7:**
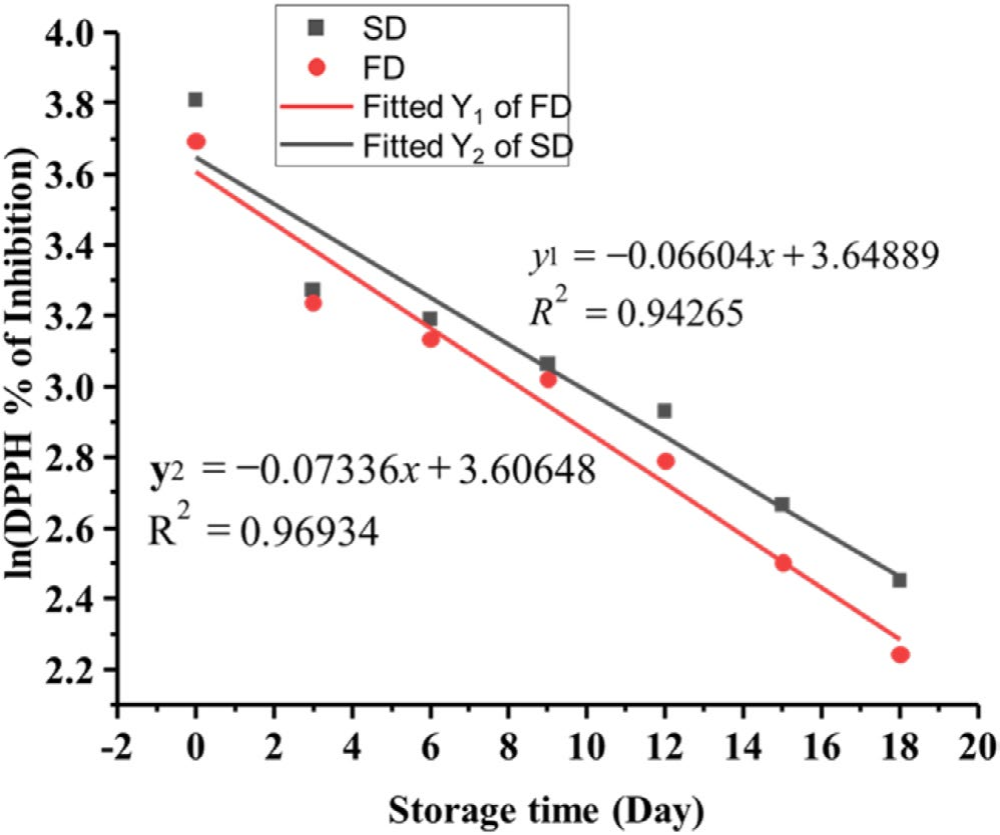
Linear curves of changes in % of DPPH scavenging values during storage in first‐order reaction.

The first step in determining shelf‐life involves identifying the degradation reaction order and fitting the degradation data to a suitable model with coefficients of determination (*R* ^2^) greater than 0.9, as shown in Table 3. The natural logarithm of the percentage of DPPH inhibition over storage time is plotted. The constant rate of reduction in DPPH radical scavenging activity (% per day), denoted as “*k*”, is derived from the slope of the regression equation of this plot. In the current study, the first‐order model was selected based on the highest *R* ^2^ values obtained from the regression analysis, as detailed in Table 3 for both samples. The linearity of the first‐order graphs for both samples is depicted in Figure 7 below.

(7) $$Y_{1}=-0.06604x+3.64889$$

(8) $$Y_{2}=-0.07336x+3.60648$$

where *Y*_1_ for spray dried microcapsule (SD) and *Y*_2_ for freeze dried powder, the slope of the equations is the rate constant of the microcapsule. Therefore, the shelf life of the microcapsule was calculated based on the following equation:

(9) $$t=\left(\ln A_{o}-\ln A_{t}\right)/k$$

where, *t* = predicted shelf life (days) ln*A*_o_ = initial % of DPPH scavenging activity before storage, ln*A*_*t*_ = value of % of DPPH scavenging activity after time *t* and *k* = rate constant.

**TABLE 3:** Constant values and zero and first‐order reaction kinetics based on % DPPH radical scavenging activities on storing at 40°C.

Parameters	Zero order		First order		Shelf life (days)	
	SD	FD	SD	FD	SD	FD
*R* ^2^	0.8	0.9001	0.94256	0.96934	21.02	18.6
*K*	1.54	1.57	0.066	0.08		

Abbreviations: FD, freeze drier; SD, spray drier.

The formula given above can be used to predict shelf life in days or months. Using this equation, the shelf life of microcapsules prepared by spray‐drying (SD) and freeze‐drying (FD) was determined to be 21 and 19 days, respectively, at storage conditions under 40°C. The results indicated a slight difference in the storage stability of SD and FD microcapsules In general, the storage stability of microcapsules largely depends on the particle size, shape, residual moisture, and encapsulation efficiency of the microparticles.

## 4 Conclusion

The essential oil of *T. schimperi* was encapsulated using spray drying (SD) and freeze‐drying (FD) techniques with gum Arabic as a coating agent. Analysis of physical and chemical characteristics of the capsules revealed that SD exhibited superior encapsulation efficiency than FD. SD powder displayed excellent surface morphology, smaller particle size, lower surface oil content, higher encapsulation efficiency, lower moisture content, high solubility index, and superior release properties compared to FD microcapsules. Both samples showed good fit with three mathematical models: zero order, Korsmeyer‐Peppas, and Higuchi, as indicated by high determination coefficients (*R* ^2^) close to 1. FD microcapsules contained higher total oil content than SD microcapsules, possibly due to the low temperature in the drying process which preserved volatile oil components better. Chemical analysis via FTIR spectroscopy indicated that both SD and FD microcapsules exhibited similar spectral bands, although FD powder showed a weaker spectrum compared to SD and gum Arabic. The storage stability degradation kinetics of both microcapsules was similar, and they displayed comparable first‐order degradation kinetics.

## Author Contributions

**Yisgedu Asres:** conceptualization (equal), formal analysis (equal), investigation (lead), methodology (equal), project administration (equal), resources (equal), visualization (equal), writing – original draft (equal), writing – review and editing (equal). **Ariaya Hymete:** conceptualization (equal), formal analysis (equal), methodology (equal), supervision (equal), visualization (equal), writing – review and editing (equal). **Habtamu Admassu:** conceptualization (equal), formal analysis (equal), methodology (equal), supervision (equal), visualization (equal), writing – review and editing (equal). **Amare Ayalew:** conceptualization (equal), formal analysis (equal), methodology (equal), supervision (supporting), visualization (equal), writing – review and editing (equal).

## Conflicts of Interest

The authors declare no conflicts of interest.

## Data Availability

The data that support the findings of this study are available from the corresponding author upon reasonable request.
